# Optimization of Process Parameters, Microstructure, and Properties of Laser Cladding Fe-Based Alloy on 42CrMo Steel Roller

**DOI:** 10.3390/ma11102061

**Published:** 2018-10-22

**Authors:** Jiang Ju, Yang Zhou, Maodong Kang, Jun Wang

**Affiliations:** 1 Shanghai Key Laboratory of Advanced High-Temperature Materials and Precision Forming, Shanghai Jiao Tong University, Shanghai 200240, China; jujiang1990@sjtu.edu.cn (J.J.); yzhou76@sjtu.edu.cn (Y.Z.); 2 School of Materials Science and Engineering, Shanghai Jiao Tong University, Shanghai 200240, China

**Keywords:** laser cladding, Fe-based powder, process parameters, microstructure, properties, mould foot roller

## Abstract

The mould foot roller is a key component of a continuous casting machine. In order to investigate the possibility of using laser cladding to repair mould foot roller, Fe-based powders and 42CrMo steel are used in this work. The laser cladding process parameters were optimized by orthogonal experiments. The chemical compositions, microstructure, properties of the cladding layer under the optimum process parameters, and substrate were systematically investigated by using optical microscopy (OM), scanning electron microscopy (SEM), energy dispersive spectroscopy (EDS), X-ray diffraction (XRD), microhardness test, wear test, and salt spray corrosion test. The results indicate that the primary factor affecting the width and depth of the cladding layer is laser power. The scanning speed also has a significant effect on the height of the cladding layer. The optimum process parameters for repairing the mould foot roller are 2 kW laser power, 4 mm/s scanning speed, and 15 g/min feeding rate of powder. Along the depth direction of the cladding layer, the microstructure of the coating gradually transforms from plane crystal, cell grains, or dendrites to equiaxed grains. The matrix is mainly martensite with retained austenite; the eutectic phase is composed of netlike M_2_B, particulate M_23_(C,B)_6_, and M_7_(C,B)_3_ phase. The hardness of the cladding layer is significantly improved, about three times that of the substrate. The weight loss of the cladding layer is just half that of the substrate. Its wear resistance and corrosion resistance have been significantly improved. The work period of the laser cladding-repaired foot roller is much longer than for the surfacing welding-repaired one. In summary, laser cladding technology can increase the life of mould foot rollers.

## 1. Introduction

Laser cladding is an advanced surface-strengthening and repair technology for the production of good metallurgical bonding coatings [1,2,3,4]. It serves as an effective and appropriate method to improve the substrate properties by forming dense coatings. The process is to melt and solidify the pre-alloyed powder onto the substrate, and the connection between the coating and the substrate is a metallurgical bond with small dilution [5]. Compared with conventional techniques such as thermal spraying or submerged arc welding (SAW), the coatings obtained by laser cladding exhibit higher surface quality and have a low dilution rate, high bonding strength with substrate, small heat-affected zone, and less material loss. Moreover, it is also a cost-saving and efficient technique, and has extensive applications in many industrial fields [6,7,8]. Therefore, laser cladding technology has attracted considerable attention.

Fe-, Ni-, and Co-based alloy powders are explored and applied more extensively and commercially in all coating materials [9,10,11,12]. Compared with other alloys, Fe-based alloy coatings have more advantages [13,14,15,16,17,18]: (1) The chemical composition and expansion coefficient are closer to the substrate (steel). Therefore, the coating can combine with the substrate well. It does not produce cracks or holes. (2) Not only is the microhardness and toughness of the Fe-based coating as good as that of the Ni-based and Co-based alloys, but the production cost of Fe-based alloy powder is much lower, which is the biggest advantage in commercial applications and large area repair. (3) The Fe-based coating, containing a large amount of corrosion-resistant elements (Cr, etc.), has superior wear resistance and corrosion resistance. (4) The existence of B and Si elements can decrease the melting temperature of the alloy and increase the fluidity of the powder. Moreover, their oxidation resistance and slagging function can protect the molten pool. As a result, it is worthwhile investigating the properties of Fe-based alloy powder.

The mould foot roller is a key component of the continuous casting machine, which plays an important role in the steel industry [19]. The performance of mould foot rollers not only directly affects the service life, but also the quality and efficiency of steel products. The working environment of mould foot rollers is very harsh, including high working temperature, wear and tear. The corrosion resistance of mould foot rollers is also a big concern because the cooling water is from seawater desalination. The 42CrMo steel cannot protect against wear and corrosion [20,21,22,23]. Therefore, it is probably a good idea to clad a layer of Fe-based alloy powder on the 42CrMo substrate. During the process of laser cladding, the cladding parameters play an important role in the size of the cladding layer [24]. The process parameters, such as laser power, powder feeding rate, laser scanning speed, laser beam spot size and shield gas flow, have significant influences on the final manufactured product. The effect of process parameters on the properties of cladding has been studied extensively [25,26,27]. However, each work requires an appropriate choice of process to satisfy its particular needs, and a systematic study is still lacking.

In this work, nine different groups of processing parameters were designed through orthogonal experimental tests. The microstructure, hardness, wear resistance, and corrosion resistance of the coating were tested. We hope to provide experimental guidance on the use of laser cladding Fe-based alloy to repair mould foot rollers.

## 2. Experimental Materials and Methods

### 2.1. Material Preparation

**Figure 1:**
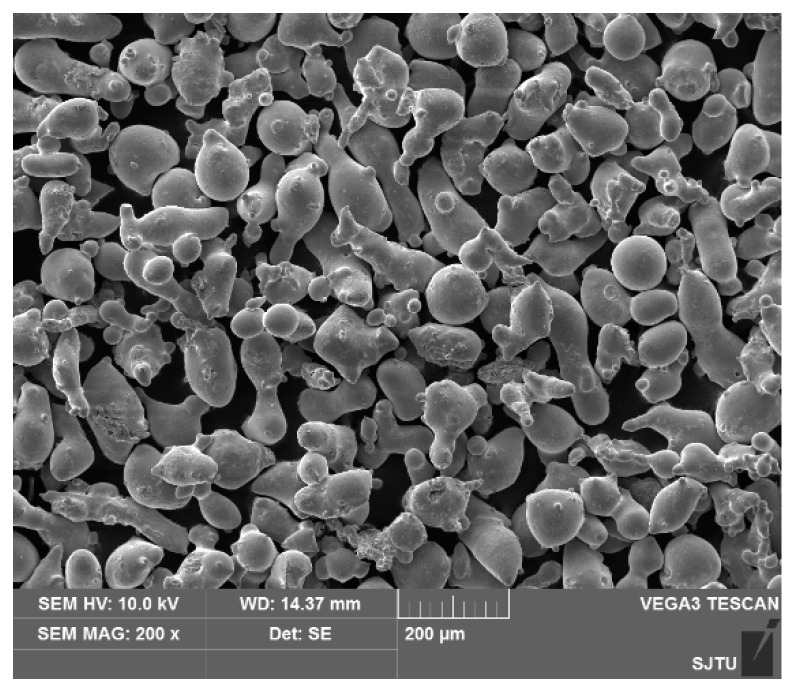
The morphology of Fe-based alloy powder.

Plates of 42CrMo steel, with dimensions of 100 mm × 50 mm × 10 mm, are used as the substrate. The cladding material is an Fe-based alloy powder whose particle size was 50~105 μm, as shown in Figure 1. The compositions of the 42CrMo steel substrate and alloy powder are given in Table 1. Before experimentation, the powder was dried in a tube furnace (OTF-1200X, HF-Kejing, Hefei, China) and mixed in a ball shaker for 12 h to achieve uniform distribution. We sanded the surface of the substrate with sandpaper and cleaned it with acetone.

**Figure 2:**
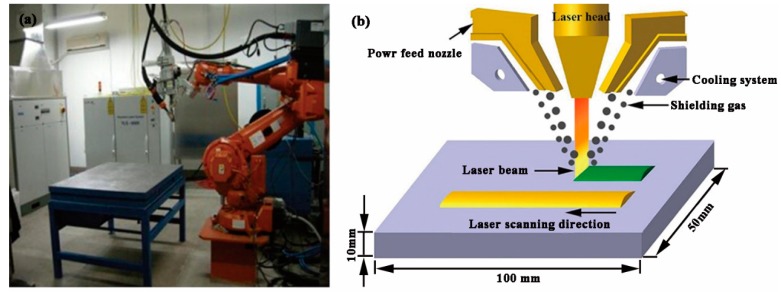
Experimental setup: (**a**) overall experimental setup; (**b**) schematic of laser cladding process.

A fiber laser is used for laser cladding in this work, with a wavelength of 1070 nm and 6 kW power. The fiber laser cladding system mainly includes an IPG YLS-6000 fiber laser (IPG Photonics Corporation, Oxford, MA, USA, 5 mm × 5 mm square spot), and a DPSF-2 powder feeding system with a coaxial and a cooling system. The energy distribution is uniform. When a laser cladding process was completed, the specimens were air-cooled to room temperature. High-purity argon gas (≥99.9%) was used as a shielding gas to protect the molten pool; the flow rate was about 15 L/min. Based on previous research, the laser cladding parameters in this work are given in Table 2. The schematic sketch of the laser cladding process is shown in Figure 2b.

### 2.2. Microstructure Observation 

All analytical specimens were sectioned by wire-electrode cutting, with a dimension of 10 mm × 10 mm × 15 mm. All specimens, after sanding and polishing, were etched in a solution of aqua regia, HCl and HNO_3_ in a volume ratio of 3:1. The height, width and depth of the layer were measured using OLYMPUS BX51 optical metallographic microscope. The microstructures of the cladding layer and substrate were examined by means of optical microscope (OM, OLYMPUS BX51, OLYMPUS, Japan) and scanning electron microscope (SEM, JSM-7800F Prime, JEOL, Osaka, Japan) with Energy-Dispersive Spectrometer (EDS, JSM-7800F Prime, JEOL, Osaka, Japan) attached. The phase components were determined by X-ray diffraction (XRD-7000S, Shimadzu, Kanagawa, Japan). Cu-Kα radiation at 40 kV and 200 mA was used as the X-ray source. The specimens were scanned in an angular 2 θ ranging from 20° to 80°. The step size was 0.2° and the collection time was 10 s [28].

### 2.3. Hardness and Wear Resistance Tests

The microhardness of the cladding layer was measured using a HV-1000 digital microhardness tester (SIOMM, Shanghai, China). In the middle of the cladding layer, 10 points, with an average interval of 0.1 mm, were selected for measurement. The average value of microhardness was calculated after getting rid of the maximum and the minimum values. The microhardness value of the sample from best laser process parameters was measured every 0.2 mm along the depth direction from coating to substrate. The test load was 500 g, with a 10 s loading time. Rockwell hardness was tested using the HR-150A-type Rockwell hardness tester (Shanghai optical instrument factory, Shanghai, China) with a load of 150 kg. The Rockwell hardness was the average value of 10 random test values from the multi-pass sample surface.

The wear resistance was tested using M-200 wear test machine (Air Times, Beijing, China). The specimen size is 10 mm × 10 mm × 12 mm. The grinding ring material is high-carbon steel GCr15 steel with quenching hardness and specifications of 60~62 HRC and φ 40 mm × 10 mm, respectively [29]. The test was performed at 20 °C for 30 min with a test load of 30 kgf at 200 rpm. The wear loss of the sample was weighed using TG328B type balance (METTLER TOLEDO, Zurich, Switzerland), whose sense and range are 0.1 mg and 0~200 g, respectively. The morphology of the worn surfaces was observed using Quanta FEG650 scanning electron microscope (FEI, Hillsboro, OR, USA).

### 2.4. Salt Spray Corrosion Test

The corrosion property of the cladding layer and substrate was tested using an FQY015 salt spray corrosion tester (Shanghai Laboratory Instrument Works, Shanghai, China). The concentration of sodium chloride solution was 5 wt %, and the spray mode was continuous. The test temperature was 35 °C. The weight of the sample was measured every 24 h using a TG328B analysis balance. The total test time was 168 h.

## 3. Results and Discussion

### 3.1. Optimization of Process Parameters

#### 3.1.1. The Macro Appearance of Laser Cladding Layer

**Figure 3:**
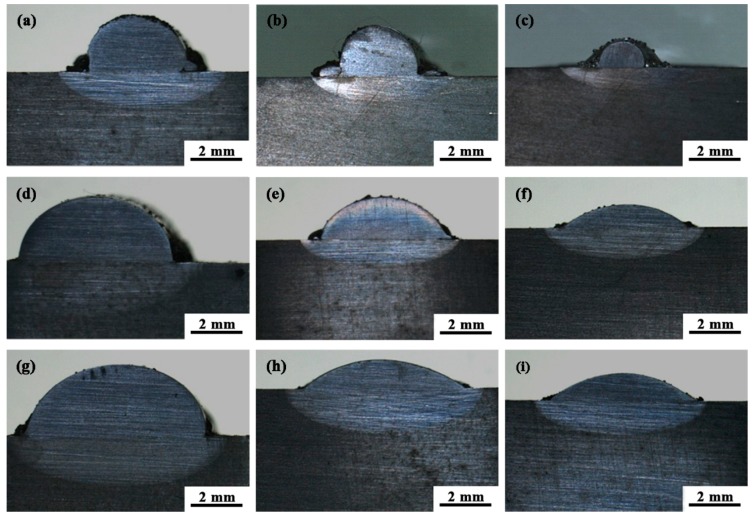
The macro morphology of cladding layer obtained by different processes: (**a**) No. 1 group; (**b**) No. 2 group; (**c**) No. 3 group; (**d**) No. 4 group; (**e**) No. 5 group; (**f**) No. 6 group; (**g**) No. 7 group; (**h**) No. 8 group; (**i**) No. 9 group.

Figure 3 represents the macro morphology of cladding layer obtained in different process. From Figure 3, the cladding layers are well formed, while the geometry size of No. 2 and No. 3 cladding layer is very small. This is mainly due to low laser power and a fast scanning speed in both groups, which means that the laser energy density is not enough to completely melt the powder. As a result, it cannot form a good metallurgical bond with the substrate. Moreover, there are obvious differences in height and width among all the cladding layers because of the different processing parameters.

**Figure 4:**
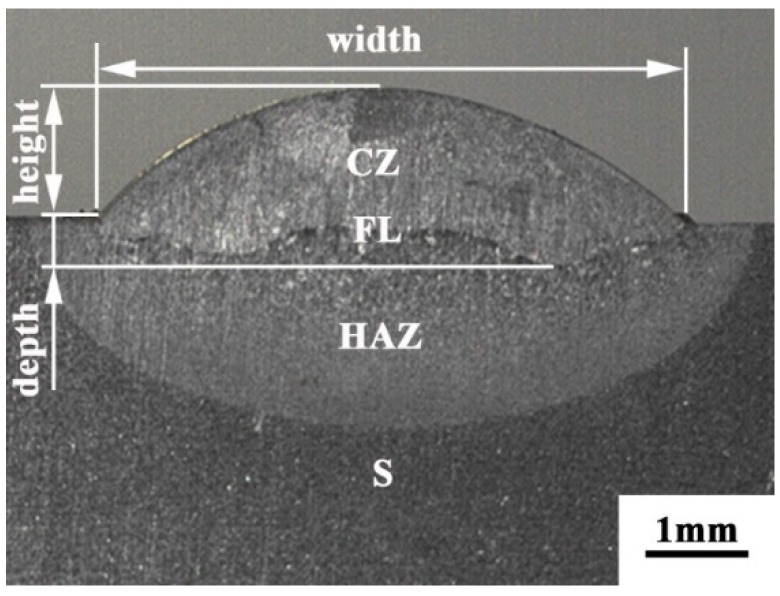
Macro-appearance of the cross section.

The morphology of the cross section of the single track cladding layer is shown in Figure 4. It can be seen from Figure 4 that the cladding layer mainly consists of a cladding zone (CZ), a fusion line (FL), the heat-affected zone (HAZ), and the substrate (S). The existence of the fusion line means that the cladding layer combined with the substrate well. We did not find cracks or porosity in the cross section, which means that the cladding layers are well formed. The test data for the height, width, and depth of the cladding layer are shown in Table 3. 

#### 3.1.2. The Analysis of Orthogonal Experiment Results

Through the analysis of orthogonal experiment data and the comprehensive comparison, complex multi-factor processing problem can be converted into a single-factor problem. The relationship and comparison between the factors and the test data can be found. The results of the range analysis data are given in Table 4. *K* value represents the average of the respective level values for each factor. *R* is the range analysis data of each factor, which is the maximum value minus the minimum value from *K*_1_ to *K*_3_ in the same column. Its size reflects the degree of influence of a factor on a certain index. The greater *R* value makes the effect of the process parameters better. 

**Figure 5:**
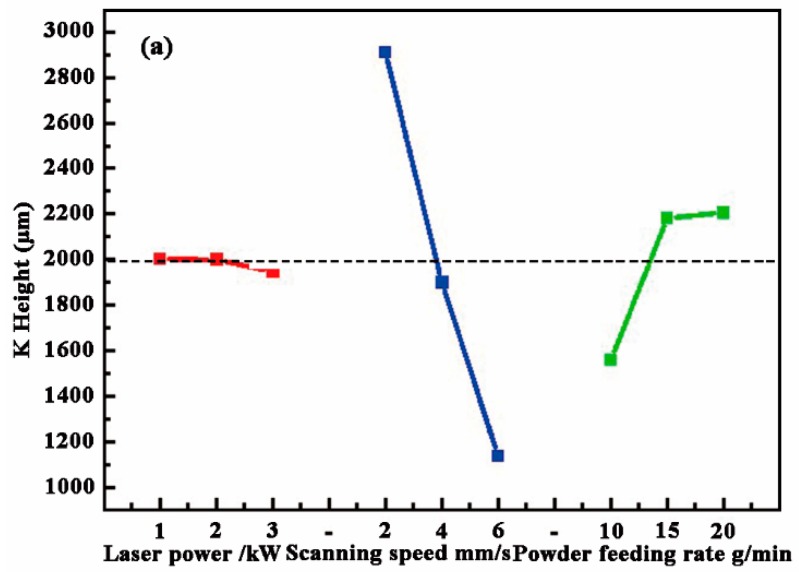

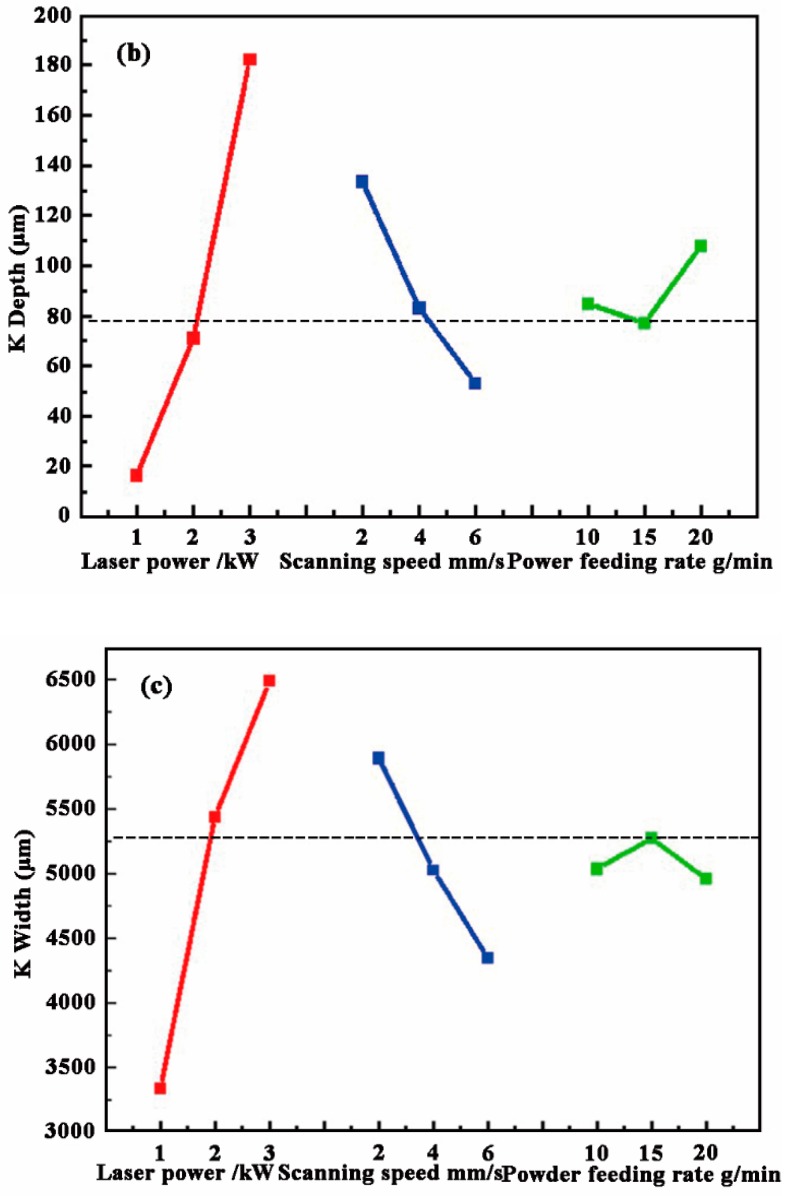
Effect of process parameters (laser power: red, scanning speed: blue and feeding rate: green) on the (**a**) height, (**b**) depth, and (**c**) width of the cladding layer.

The *K* values in Table 4 are shown in a line chart in Figure 5. Combined with the results of the range analysis data, it can be seen that the scanning speed has the greatest impact on the height of the CZ. As the scanning speed decreases, more powder is melted into the pool, which contributes to increasing the height. The biggest factor affecting the depth is the laser power. The other two variables have little influence on the depth. The main reason is that, as the power increases, the energy input gradually increases, which makes more S melt into the pool, leading to an increase of depth after rapid solidification. The *R* value of the laser power to width is the biggest, which also demonstrates that laser power has a major effect on the width. The second is the scanning speed and the feeding rate of powder. The increase in laser power causes the longer time of the molten pool, which results in an increase of the width. According to the actual repair needs of the foot roller, we need to obtain a CZ with the height of about 2 mm. Hence, the target line was set at 2000 μm in the ordinate scale in Figure 4a. It can be seen that the 2 mm CZ could be obtained when the laser power, scanning speed, and powder feeding rate were 1–2 kW, 4 mm/s, and 15 g/min, respectively. Based on the height, it is desirable that the width of the CZ was larger while the depth of the CZ becomes smaller. Therefore, the minimum depth and maximum width were taken as target values. The results are shown in Figure 5b,c. Considering the best value of the three factors, the optimum process parameters for repairing the foot roller are 2 kW laser power, 4 mm/s scanning speed, and 4 mm/s powder feeding rate. 

### 3.2. Microstructure and Hardness of the Cladding Layer

#### 3.2.1. Microstructure and Phase Analysis

**Figure 6:**
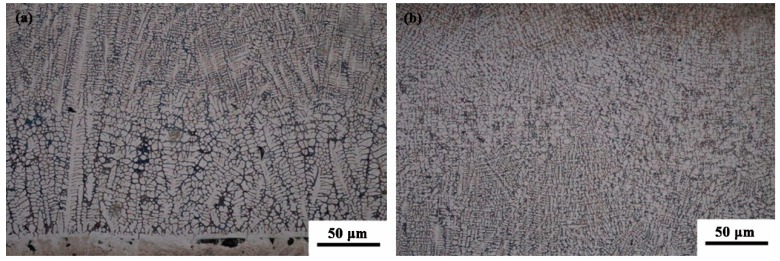
The morphology of the cladding layer: (**a**) bottom; (**b**) middle.

Figure 6 represents the microstructure of the bottom and middle part of the CZ. From Figure 6, the microstructure, close to the fusion line, is a planar structure. As the distance from the fusion line increases, the microstructure of the CZ changes to cell grains, as shown in Figure 6a. When the distance from the fusion line further increases, the microstructure gradually changes to dendrites and equiaxed grains.

It is known that the ratio of *G*/*R* determines the microstructure of the CZ, where *G* is the temperature gradient, and *R* is the solidification rate [30]. At the bottom of the molten pool, the *G* value is high while the *R* value is very low, which means that the *G*/*R* value is relatively large near the fusion line, and the plane interface is unstable due to small constitutional super-cooling. So a dendritic structure is formed that is nearly perpendicular to the fusion line, as shown in Figure 6a. However, with the distance from the fusion line increasing, the *G* and *R* values gradually decrease and increase, respectively, so the value of *G*/*R* decreases gradually from the bottom to the surface of the laser molten pool. Therefore, at the middle of the molten pool, the value of *G*/*R* is low and the constitutional supercooling becomes larger, which leads to the formation of an equiaxed structure. 

**Figure 7:**
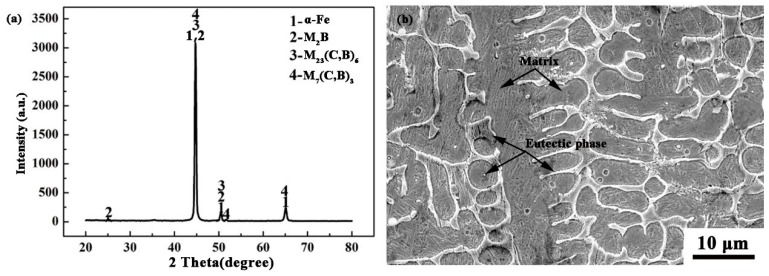
The XRD result and SEM morphology of the dendrite at the middle: (**a**) XRD result; (**b**) SEM morphology.

**Figure 8:**
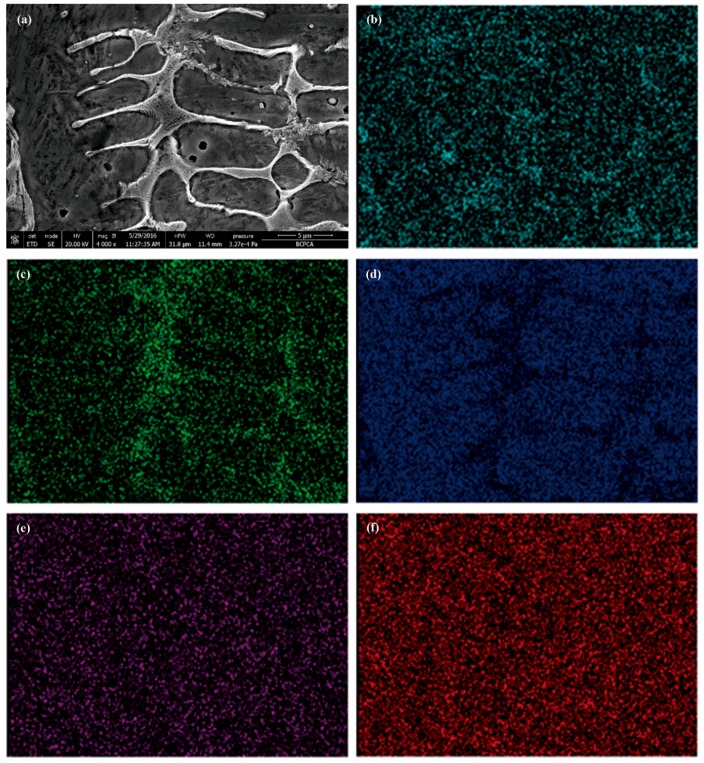
The EDS surface scan result: (**a**) SEM photo; (**b**) C; (**c**) Cr; (**d**) Fe; (**e**) Ni; (**f**) Si.

From Figure 7b, the microstructure consists of a matrix and a eutectic phase. The XRD results in Figure 7a show that the matrix is mainly α-Fe; the eutectic phase is mainly composed of M_2_B, M_23_(C,B)_6_ and M_7_(C,B)_3_ (M = Fe, Ni). Lei also found similar phases in the NiCrBSi composite coating [30,31]. According to the results of the EDS mapping (as shown in Figure 8) and XRD, the eutectic M_2_B has a reticular structure and gathers in the grain boundary. The M_23_(C,B)_6_ is a particulate or short rod-like phase, which was dispersed uniformly in the matrix. M_7_(C,B)_3_ and M_23_(C,B)_6_ are formed by the eutectoid reaction of γ-Fe with different C content. Since the solubility of B in γ-Fe and α-Fe is very low, after adding excess B element, the segregation at grain boundary makes a part of B element form M_2_(B,C), and the rest of B element is dissolved into M_7_C_3_, forming M_7_(C,B)_3_ [32]. Cr has a higher tendency to form carbides than Fe, so it is easier to form M_7_C_3_ [33]. From Figure 8, the microhardness of the cladding layer is more than 500 HV; Lin also finds that the hardness of the Fe-based alloy coating ranges from 500 HV_0.2_ to 700 HV_0.2_ [34], which indicates that the matrix may be martensite. The elevated Cr and Mo content can make *M*_s_ and *M*_f_ be reduced, which forms retained austenite at room temperature [35]. Therefore, the retained austenite also exists in this CZ, although the XRD results are not shown. It was noted that carbides and borides play an important role in strengthening the matrix and increasing the wear resistance of the CZ.

#### 3.2.2. Hardness

**Figure 9:**
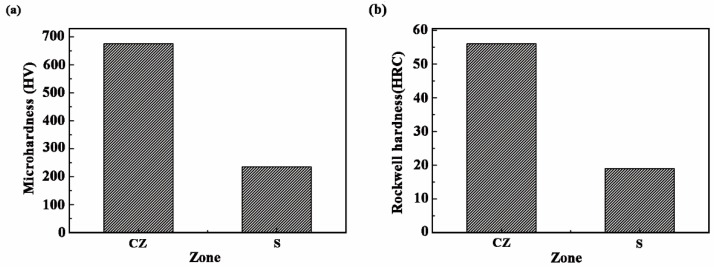
(**a**) Microhardness and (**b**) Rockwell hardness of the CZ and S.

**Figure 10:**
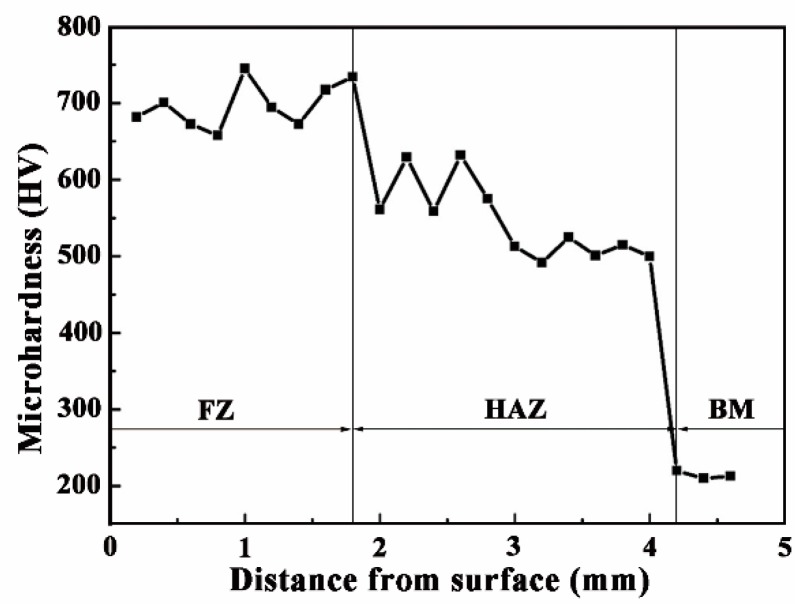
The distribution of microhardness along the depth direction.

Figure 9 shows the microhardness and Rockwell hardness of CZ and S. The microhardness distribution of the CZ and S of a single track specimen through the depth are shown in Figure 10. From Figure 9, the microhardness of the CZ is about three times that of the S. The Rockwell hardness of the CZ also is about three times that of the S. From Figure 10, the curve indicates a ladder shape, in which the average hardness of the FZ is about 700 HV. The hardness of HAZ reaches about 550 HV, while the microhardness of the unaffected S is 220 HV. The microhardness of the CZ is much higher than that of the S. The microhardness of HAZ is higher than that of BM, because part of the hard phase diffuses to the HAZ. Meanwhile, rapid cooling at high temperature plays the role of quenching, which also improves the microhardness. Although the microhardness of the Fe-based alloy coating is lower than that (>1000 HV_0.2_) [34] of 5 wt % Cr_3_C_2_ reinforced Fe-based composite coating, but the cost of the Fe-based alloy coating is cheaper.

### 3.3. Wear Resistance and Corrosion Resistance

**Figure 11:**
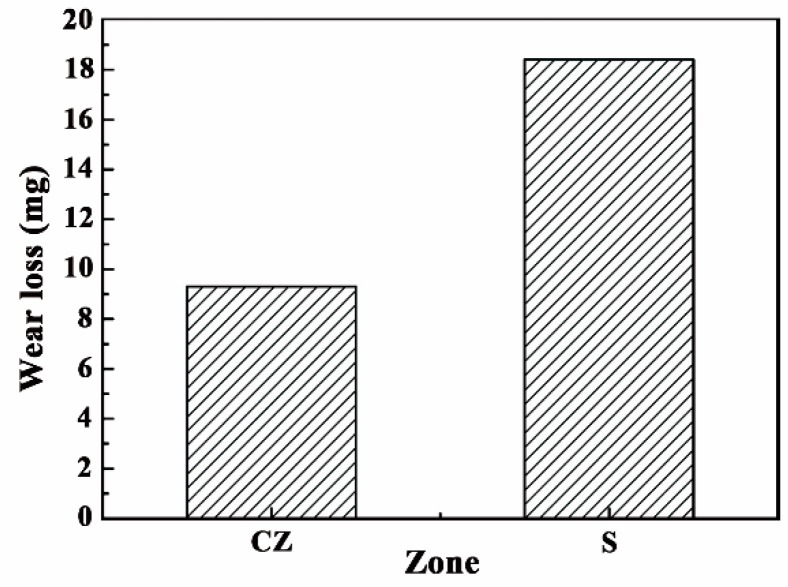
The wear loss of the CZ and S.

**Figure 12:**
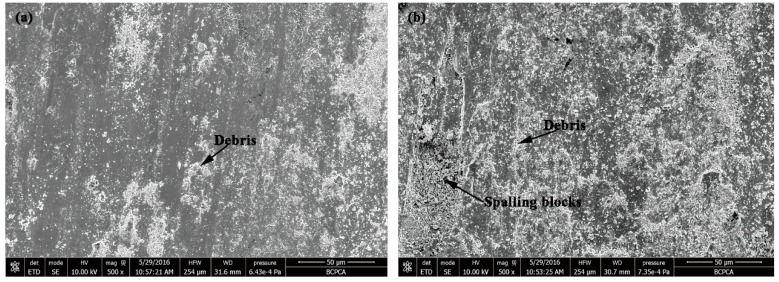
The SEM images of wear surface: (**a**) CZ; (**b**) S.

The wear loss of CZ and S is shown in Figure 11. As can be seen, the wear loss of the CZ is only half that of S under the same wear conditions, which means that the wear resistance of the CZ is obviously better than that of S. Figure 12 represents the wear surface SEM images of the CZ and S. From Figure 12a, there are some fine particles, debris, and small scratches on the surface of the CZ. In comparison, a large amount of particles and debris, as well as large spalling blocks, can be found on the surface of S, as shown in Figure 12b.

**Figure 13:**
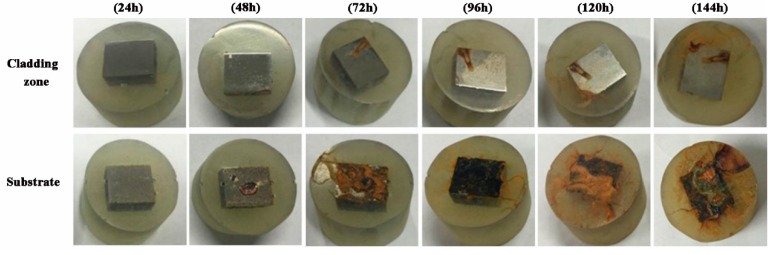
The surface macroscopic morphology of the CZ and S.

**Figure 14:**
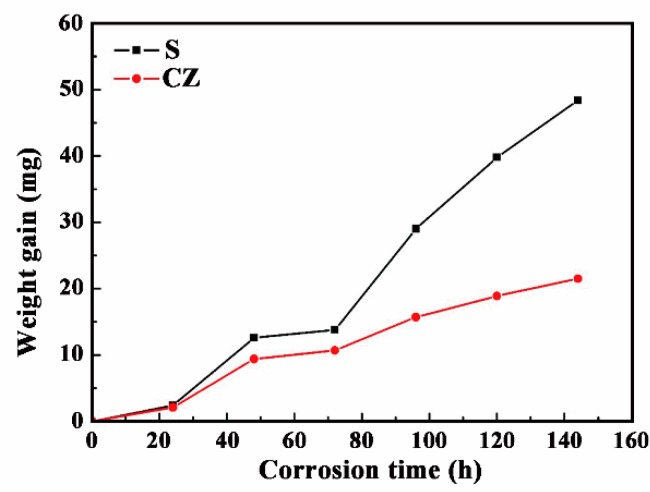
The weight gain comparison of CZ and S.

Figure 13 shows the surface macroscopic morphology of the CZ and S after a salt spray corrosion test. The weight gain comparison of the two specimens is displayed in Figure 14. It can be seen that the corrosion resistance of CZ is better than that of S. During the experimental process, the CZ has little oxidation area and weight gain, which is also in stable condition after 96 h. The weight of the substrate increases continuously, and the erosion area becomes larger until the entire surface is seriously oxidized and corroded. The colour of the corrosion layer gradually changed from brown to black, which means that the corrosion degree is getting deeper.

### 3.4. Discussion

The microhardness and Rockwell hardness of the cladding layer are much higher than those of the substrate. The main reason is that the characteristics (rapid heating and cooling) of laser cladding can refine the grain size, which plays the role of fine grain strengthening. Secondly, a large number of hard phases with high hardness (M_2_B (1300 HV), M_7_(C,B)_3_ (1300~1800 HV), and M_23_C_6_ (1450 HV)) precipitates from the matrix during the solidification process [36,37], which produces a second phase strengthening effect. Moreover, the matrix phase of the cladding zone is martensite (480~560 HV) [29], which also improves the hardness of the cladding layer.

The wear resistance of the cladding layer is obviously better than that of the substrate. The microstructure of the cladding layer contains the martensite matrix and hard phase. A martensite matrix with high hardness can provide good support to the carbides and borides along the grain boundary. Hence, it is difficult for the hard phase to fall off during the wearing process. Conversely, these hard phases with high hardness can also provide good protection to the matrix phase [38], forming a wear-resistant framework during the wearing process, which in turn reduces the wear loss of the matrix. Hence, the cladding layer has excellent wear resistance. The hard phase gradually protrudes and gets worn out after shedding the matrix phase. A small amount of debris can be formed during repeated wearing, as shown in Figure 12a. The matrix phase, martensite, also has high hardness. It can protect the hard phases from being pushed over and pressed into the matrix when they emerge in the wear process. Therefore, the scratches are small and shallow. 

Different from the cladding layer, there are lots of particles, debris, and large spalling blocks on the surface of the substrate, as shown in Figure 12b. The matrix of the substrate has low hardness. The matrix is susceptible to wear at the beginning; after the hard phase explodes, it get peeled off into particles and debris during the wearing process. The falling hard phase can easily adhere to the grinding ring in the form of small pieces due to the adhesion effect, which cut into the matrix.

In corrosion tests, due to the humid environment, Fe is oxidized to form Fe_2_O_3_ and adheres to the surface of the sample in two specimens. The presence of NaCl also accelerates the oxidation process. Since the matrix of the cladding layer is fine martensite and the solid solution contains antioxidant elements including Cr (16 wt %), the cladding layer is similar to martensitic stainless steel. Therefore, its corrosion resistance is significantly better than the substrate. The good corrosion resistance of the cladding layer is beneficial to provide protection to the substrate, and can also improve its service life in industrial applications.

### 3.5. Comparison under Service Condition

**Figure 15:**
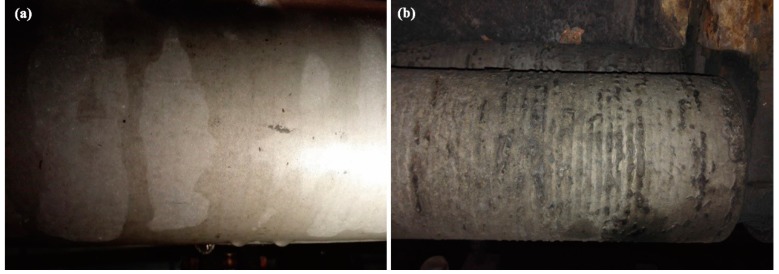
The surface comparison of two kinds of foot roller after three work periods: (**a**) repaired by laser cladding; (**b**) repaired by resurfacing welding.

In order to test and verify the effect of laser cladding, two kinds of foot roller were used. One was repaired by resurfacing welding; the other was repaired by laser cladding of the optimized process parameters and Fe-based powder. After three working periods, the surface morphologies of the cladded layer and the substrate were as shown in Figure 15. It is obvious that the condition after laser cladding looks much better than after resurfacing welding. Lots of cracks, wear, corrosion, and deformation can be seen in Figure 15b, which means that the foot roller could not be used any more. On the contrary, only a few defects appeared in Figure 15a.

## 4. Conclusions 

In this paper, laser cladding technology was used to repair a mould foot roller. The article is in two parts, including the optimization of process parameters and research into the microstructure and properties of the cladding layer under the optimum process parameters. The main research results were as follows:

(1) The laser power has the primary effect on the width and depth of the cladding layer. The scanning speed has a significant effect on the height. The optimum process parameters for repairing the mould foot roller are 2 kW laser power, 4 mm/s scanning speed, and 15 g/min powder feeding rate.
(2) Along the depth directions, the crystal styles of the cladding zone gradually transformed from planar, dendritic, to equiaxed grain. The microstructure of the cladding layer consisted of matrix phase (martensite) and eutectic phase (netlike M_2_B, particulate M_23_(C,B)_6_, and M_7_(C,B)_3_).
(3) The microhardness and Rockwell hardness of the cladding layer are about three times that of the substrate. The weight loss of the cladding layer is just half that of the substrate.
(4) The wear resistance and corrosion resistance of the cladding layer are better than those of the substrate. The excellent wear resistance is mainly due to the existence of martensite and eutectic phase with high hardness.

## Figures and Tables

**Table 1:** Compositions of the alloy powder the substrate (wt %).

Element	C	Cr	Ni	Mo	Mn	Si	B	Co	Fe
Alloy powder	0.2	16.0	2.5	0.5	-	0.75	1.0	0.5	Bal.
42CrMo steel	0.38	0.90	-	0.19	0.60	0.17	-	-	Bal.

**Table 2:** The different parameters of orthogonal experiment groups.

Group Number	No. 1	No. 2	No. 3	No. 4	No. 5	No. 6	No. 7	No. 8	No. 9
Laser power (kW)	1	1	1	2	2	2	3	3	3
Scanning speed (mm/s)	2	4	6	2	4	6	2	4	6
Feeding rate (g/min)	10	15	20	15	20	10	20	10	15

**Table 3:** Size of all nine groups of samples.

Number	No. 1	No. 2	No. 3	No. 4	No. 5	No. 6	No. 7	No. 8	No. 9
Height (μm)	2506.8	2335.0	1961.1	2914.2	2140.4	944.4	3311.9	1228.8	1296.5
Depth (μm)	22.8	15.9	10.8	115	50.6	48	262.9	183.8	100.9
Width (μm)	4175.6	3618.8	2226.9	6189.6	5331.2	4805.4	7323.5	6137.6	6009.6

**Table 4:** Range analysis data sheet.

	Laser Power (kW)			Scanning Speed (mm/s)			Powder Feeding Rate (g/min)		
	Height (μm)	Depth (μm)	Width (μm)	Height (μm)	Depth (μm)	Width (μm)	Height (μm)	Depth (μm)	Width (μm)
*K*_1_	2003.1	16.5	3340.4	2911.0	133.6	5896.2	1560.0	84.9	5039.5
*K*_2_	1999.7	71.2	5442.1	1901.4	83.4	5029.2	2181.9	77.3	5272.7
*K*_3_	1945.7	182.5	6490.2	1136.1	53.2	4347.3	2206.6	108.1	4960.5
*R*	57.4	166.0	3149.8	1774.9	80.4	1548.9	646.6	30.8	312.2
